# Data-driven classification and design of bioactive cellulose acetate electrospun films for active food packaging

**DOI:** 10.1371/journal.pone.0352581

**Published:** 2026-06-30

**Authors:** Ahmad Rabbani, Anuj Niroula, Muhammad Z. Iqbal, Sajid Maqsood, Akmal Nazir

**Affiliations:** 1 Department of Food Science, College of Agriculture and Veterinary Medicine, United Arab Emirates University, Al Ain, United Arab Emirates; 2 Department of Chemical and Petroleum Engineering, College of Engineering, United Arab Emirates University, Al Ain, United Arab Emirates; Amity University Noida, INDIA

## Abstract

The development of bio-based food packaging materials is increasingly important for reducing reliance on petroleum-based plastics. For practical packaging use, these materials must also achieve suitable mechanical performance, including strength, flexibility, and stiffness. This study reports the fabrication of bioactive cellulose acetate (CA) films containing aloe vera gel (AG) and lemongrass essential oil (EO) using electrospinning, aimed at developing mechanically robust films with potential relevance to active food packaging applications. Mechanical performance was systematically assessed across 11 formulations generated by a Latin Hypercube Design. Tensile strength (TS), elongation at break (EAB), and Young’s modulus (YM) were measured, and response surface methodology was employed to model the effects of CA, AG, and EO, including their interactions. CA emerged as the primary determinant, improving TS and YM but reducing EAB at higher concentrations. AG functioned as a natural plasticizer, enhancing flexibility while lowering stiffness, whereas EO showed conditional effects, providing limited plasticization at low concentrations and reduced mechanical integrity when combined with elevated CA or AG concentrations. Trade-offs between the measured properties emphasized the role of interactions rather than single components. Films were subsequently classified into flexible, balanced, and rigid categories based on the experimental thresholds, linking formulations with potential packaging applications and providing a basis for rational design of biodegradable packaging materials.

## 1. Introduction

The growing demand for sustainable food packaging has accelerated research into biodegradable materials that can replace conventional petroleum-based plastics [1]. Traditional plastics offer durability and versatility, but persist in the environment, contributing to serious ecological concerns and waste management challenges [2]. As a result, biopolymer-based films have gained increasing attention as renewable, biodegradable, and often compostable alternatives [3]. Materials such as starch, chitosan, polylactic acid (PLA), and cellulose derivatives have been widely explored for their potential to meet packaging requirements while reducing environmental impact [4]. Among these, cellulose acetate (CA) is particularly attractive due to its renewable origin, excellent film-forming ability, good transparency, and favorable barrier properties [5]. These features, combined with its established history of use in packaging-related materials, make CA a promising candidate for the development of next-generation bio-based packaging films [5,6].

Despite its favorable attributes, CA suffers from inherent limitations that restrict its direct use in packaging applications. Pure CA films are typically brittle, displaying limited extensibility and reduced toughness, which compromises their suitability for flexible packaging formats such as wraps or seals [7]. Achieving the right balance of mechanical properties (tensile strength, elongation at break, and stiffness) is essential, as packaging films must withstand the handling stresses while maintaining product integrity [8]. To address these limitations, researchers have explored blending CA with plasticizers, natural polymers, and bioactive compounds [9–12]. Incorporating additives such as aloe vera gel or essential oils not only improves flexibility and mechanical performance but also introduces additional functionalities, including antioxidant or antimicrobial activity [13–15]. However, the impact of these modifications on the overall mechanical profile of CA films remains complex, as interactions between the base polymer and additives could lead to trade-offs between strength, stiffness, and flexibility.

Most studies on CA-based and other biopolymer films have focused on reporting mechanical or functional properties in isolation, often comparing average values across different formulations [16–18]. While such approaches provide useful baseline data, they fall short in translating material performance into practical packaging applications. For example, a film with high tensile strength but low elongation may be unsuitable for wrapping yet valuable for rigid tray applications, but such distinctions are rarely made. Without a systematic framework, it is difficult to classify films according to their mechanical performance or to derive formulation guidelines tailored to specific packaging needs. Moreover, the complex and often nonlinear interactions among polymer concentration, plasticizers, and bioactive additives highlight the need for data-driven methodologies that can capture these relationships, move beyond simple trial-and-error, and provide predictive tools for material design.

Although statistical tools such as response surface methodology (RSM) have been increasingly applied to optimize film formulations [19–21], few studies have extended these approaches toward a classification framework that links mechanical performance to functional packaging categories [22,23]. On the other hand, space-filling Latin hypercube designs offer a promising pathway for efficiently exploring multidimensional formulation spaces, enabling robust modeling of mechanical trade-offs across diverse film compositions [24]. Prior research has largely focused on single-property optimization, overlooking the fact that packaging applications often demand a balance between strength, flexibility, and stiffness rather than the maximization of one attribute alone. Moreover, while the incorporation of bioactive components such as AG or EOs has been explored for their functional benefits [25,26], their role in systematically tuning the mechanical behavior of CA films has not been comprehensively assessed. To date, no study has established a data-driven classification system that categorizes CA films into distinct functional types, such as flexible wraps, balanced multipurpose films, or rigid protective packaging, and subsequently uses this framework to guide design decisions. This highlights a clear research gap in linking formulation-dependent mechanical behavior of CA-AG-EO films with practical mechanical classification for packaging design.

The present study addresses this gap by investigating the mechanical performance of bioactive cellulose acetate films incorporating AG and EO, and by developing a data-driven framework for their mechanical classification and design. Mechanical properties were systematically evaluated in terms of tensile strength, elongation at break, and Young’s modulus, and statistical modeling was employed to capture the influence of formulation factors and their interactions. Based on these results, films were classified into flexible, balanced, and rigid categories, reflecting their suitability for different packaging applications such as wrapping, multipurpose use, or rigid protective formats. This classification framework provides a practical basis for tailoring film formulations to meet diverse mechanical requirements in sustainable packaging. As this work was intended as an initial mechanical design study, functional validation and additional characterization were beyond the present scope.

## 2. Materials and methods

### 2.1. Materials

All reagents used in this research were of analytical grade. Cellulose acetate (molecular weight 50,000 g/mol), acetic acid, and glycerol were procured from Sigma-Aldrich (USA). Lemongrass essential oil (EO) was generously supplied by the Central Institute of Medicinal and Aromatic Plants (CIMAP), India and was used from the same supplied batch throughout the study. Fresh aloe vera leaves were harvested from the Al Maqam campus of the United Arab Emirates University (UAEU) in Al Ain, UAE. The gel was manually extracted, blended, and then filtered using a strainer to remove suspended particles before being stored at 4 ± 1 °C to preserve its bioactivity for later use [27]. The same batch of freshly extracted aloe vera gel was used for all formulations to minimize compositional variability.

### 2.2. Experimental design

A space-filling Latin Hypercube Design (LHD) was employed to investigate the effect of three continuous formulation variables: cellulose acetate (CA, 3–6%), aloe vera gel (AG, 0–25%), and lemongrass essential oil (EO, 0–3%), with all concentrations expressed as % w/v. Space-filling designs ensure uniform coverage of the design space, reducing bias and model uncertainty while strengthening validation and verification [28]. The LHD was generated using JMP Pro 15 (SAS Institute Inc., Cary, NC, USA) to efficiently cover the multidimensional design space while minimizing clustering of design points. A total of 11 experimental runs were obtained (Table 1), with factor ranges selected based on preliminary screening and literature reports to ensure film-forming ability and stability. All experimental runs were randomized to reduce bias.

**Table 1:** Experimental runs generated by the Latin Hypercube Design showing the formulation levels of cellulose acetate, aloe vera gel, and lemongrass essential oil.

Experimental runs	Cellulose acetate (CA, %)	Aloe vera gel (AG, %)	Lemongrass essential oil (EO, %)
1	5.7	10	0.3
2	3	5	0.6
3	5.1	25	1.2
4	5.4	2.5	2.4
5	3.9	7.5	3
6	3.6	17.5	0
7	4.8	20	2.7
8	3.3	22.5	2.1
9	4.2	12.5	1.5
10	4.5	0	0.9
11	6	15	1.8

### 2.3. Preparation of electrospinning solutions

The different formulations listed in Table 1 were prepared by dissolving the specified amounts of cellulose acetate in acetic acid. The solution was stirred continuously at 80 °C overnight to ensure complete polymer dissolution. Glycerol (2% w/v) was added to all formulations as a plasticizer based on preliminary screening and previous reports on glycerol-plasticized biopolymer films [29,30]. Following the stirring phase, the heating was discontinued, and the solution was allowed to cool to room temperature (~20 °C) in order to preserve the bioactivity of subsequent bioactive compounds. Once cooled, EO and freshly extracted AG were sequentially incorporated into the CA solution and stirred for an additional 30 minutes to ensure homogeneity. Since EO is known to be light-sensitive, the solution was immediately wrapped in aluminum foil and stored at room temperature until electrospinning.

### 2.4. Electrospinning setup and film fabrication

**Fig 1:**
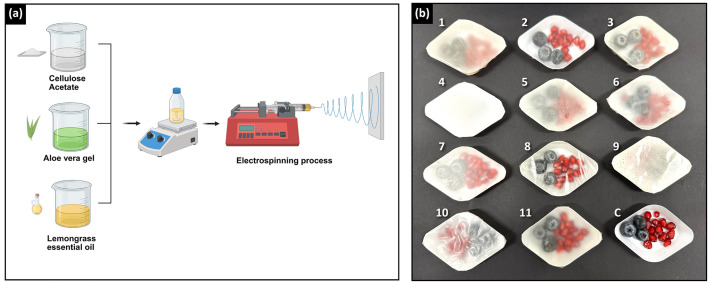
(a) Schematic of the electrospinning process used to fabricate bioactive cellulose acetate films incorporating aloe vera gel and lemongrass essential oil. (b) Visual presentation of the 11 films produced according to the experimental runs listed in Table 1, shown as coverings on food trays. The label “C” denotes the control tray without film coverage.

Electrospinning conditions were selected based on preliminary trials to obtain stable spinning and continuous film formation. Subsequently, the prepared spinning solution was loaded into a 50 mL syringe equipped with a 20-gauge metallic blunt-end needle (inner diameter: 0.584 mm; outer diameter: 0.889 mm). Electrospinning was carried out using an electrospinning unit (EF300, SKE Research Equipment, Italy). The process was conducted under ambient conditions (20 °C) in a fume hood. The applied voltage was maintained at 25 kV, with a working distance of 12 cm between the needle tip and the grounded rotating collector. A constant flow rate of 10 mL/h and a mandrel rotation speed of 400 rpm were maintained throughout the 6-hour spinning duration. To ensure consistent film morphology, the needle tip was periodically cleaned to prevent clogging due to the accumulation of viscous solution, which can disrupt jet stability and inhibit continuous formation. The electrospun CA films were collected on aluminum foil and stored at refrigerated temperature (4 °C) for further analyses. The electrospinning setup and the visual appearance of the fabricated films when applied as food-tray coverings are shown in Fig 1.

### 2.5. Characterization of the films

The thickness of each CA film was measured at five randomly selected points using a digital micrometer. Mechanical properties, including tensile strength (TS), elongation at break (EAB), and Young’s modulus (YM), were evaluated using a CT-3 texture analyzer (Brookfield, USA). For testing, the films were cut into strips measuring 80 × 15 mm and mounted on a TA-DGA dual-grip fixture. The test was performed at a crosshead speed of 0.5 mm/s with an initial trigger force of 5.0 g. Data acquisition and analysis were conducted using Texture-Pro CT software (Version 1.3, Build 15). TS was expressed in MPa by converting stress values (Eq. 1), YM was obtained from the slope of the linear (elastic) portion of the stress–strain curve (Eq. 2), and EAB was determined using the percentage increase in length at break relative to the original length of the film (Eq. 3).


$$\begin{array}{c} \text{TS }(\text{MPa})=\frac{\text{Load at break }(\text{N})}{\text{Original width }(\text{mm})\;\times \text{ Original thickness }(\text{mm})} \end{array}$$ (1)

$$\begin{array}{c} \text{YM }(\text{MPa})=\frac{\text{Stress}}{\text{Strain}}(\text{Within the elastic region}) \end{array}$$ (2)

$$\begin{array}{c} \text{EAB }(\%)=\frac{\text{Length at break }({\text{L}}_{\text{b}})-\text{Original length }({\text{L}}_{\text{o}})}{{\text{L}}_{\text{o}}}\times \text{100} \end{array}$$ (3)

### 2.6. Response surface modeling and statistical analysis

The mechanical property data (TS, EAB, YM) were first subjected to one-way ANOVA followed by Tukey’s HSD test to determine significant differences among film formulations at p < 0.05, using triplicate measurements for each formulation run. A second-order polynomial response surface model was then fitted for each property as a function of CA, AG, and EO, including linear, quadratic, and two-factor interaction terms. Model adequacy was evaluated using the coefficient of determination (R^2^), adjusted R^2^, and root mean square error (RMSE). The relative influence of formulation variables was assessed using Pareto charts of absolute standardized regression coefficients, calculated as β·SD(x)/SD(y) after excluding the intercept. Contour plots were generated to visualize the combined effects of factor pairs (CA–AG, CA–EO, AG–EO) on each response, with the third factor fixed at its median level. Predicted values from the fitted models were further compared with experimental results and used to classify films into functional categories (flexible, balanced, rigid) based on pre-defined mechanical property thresholds. All statistical analyses were performed in Python (Statsmodels/Matplotlib), and results are reported as mean values from replicate runs.

## 3. Results and discussion

### 3.1. Mechanical properties and experimental observations

**Fig 2:**
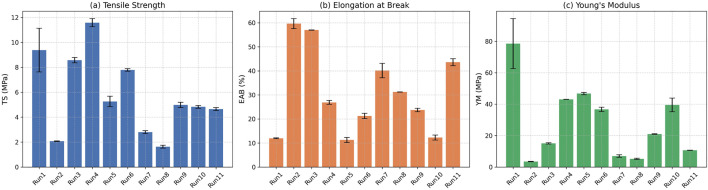
(a) Tensile strength (TS), (b) elongation at break (EAB), and (c) Young’s modulus (YM) of films (mean  ±  SD, n  =  3).

The mechanical properties of the films, expressed as tensile strength (TS), elongation at break (EAB), and Young’s modulus (YM), are presented in (Fig 2a–c). Descriptive statistics (mean ± SD) highlighted wide variability among the runs. One-way ANOVA confirmed that TS, EAB, and YM differed significantly across the 11 formulations (p < 0.001 in all cases). These comparisons indicate run-to-run differences among formulations, while the effects of individual formulation factors were interpreted using the RSM analysis described in Section 3.2.

TS values ranged from as low as ~1.6 MPa in Run 8 to ~11.6 MPa in Run 4. According to Tukey’s HSD test, high-strength films (Runs 3–4) were statistically distinct from low-strength films (Runs 2, 7, 8, and 11). Intermediate groups, such as Runs 5, 6, 9, and 10, clustered differently, reflecting the influence of compositional balance. EAB also varied significantly, from ~11% in Run 5 to ~60% in Run 2. Runs 2 and 3 formed a distinct group of highly extensible films, while Runs 1, 5, and 10 clustered as brittle films with minimal extensibility. Several other runs (6, 7, 8, 9, 11) occupied intermediate positions, demonstrating that flexibility is strongly formulation-dependent. YM spanned from as low as ~3.5 MPa in Run 2 to ~78.5 MPa in Run 1, again with significant differences among runs. Stiff films (Runs 1, 4, 5, 10) contrasted sharply with flexible runs such as 2, 7, and 8, while intermediate modulus values were observed in Runs 3, 6, 9, and 11.

These results confirm that each run followed a distinct pattern across the three mechanical parameters. Some formulations achieved high strength but at the cost of extensibility (e.g., Run 4), while others were highly extensible but mechanically weak (e.g., Run 2). This shows that no single formulation simultaneously provides optimal values for all three properties. Therefore, a modeling-based approach (Section 3.2) is needed to better explain the role of each ingredient and to guide the design of films with a suitable balance of strength, stiffness, and flexibility.

The variability in TS, EAB, and YM across formulations reflects the competing roles of CA, AG, and EO in defining film mechanics. The TS values observed in this study (1.6–11.6 MPa) fall within the lower–intermediate range reported for cellulose acetate films and their blends, which typically span 2–30 MPa depending on plasticizer content and processing. The limited extensibility of some formulations (EAB ≤ 15%) is also consistent with the brittle nature of neat CA films documented previously [31]. By contrast, formulations incorporating higher levels of AG or optimized CA–EO ratios demonstrated markedly improved flexibility, as the bioactive agents disrupt polymer chain packing and reduce intermolecular interactions and hydrogen bonding within the CA matrix, thereby lowering stiffness and enhancing extensibility, highlighting the potential of bioactive additives to overcome the rigidity of unmodified CA matrices [32]. Comparable trends have been noted in polysaccharide- and protein-based films, where natural plasticizers increase chain mobility and reduce stiffness, but often at the cost of tensile strength [33]. These results confirm that mechanical performance in biopolymer films is strongly formulation-dependent and emphasize the necessity of balancing strength, stiffness, and extensibility for practical packaging use.

### 3.2. Modeling the effect of formulation factors (RSM analysis)

To better understand how the formulation components influenced the mechanical performance of the films, the RSM approach was employed, as described in Section 2.6. Second-order polynomial models were fitted using CA, AG, and EO as independent variables to describe the variation in TS, EAB, and YM, accounting for linear, quadratic, and interaction effects. The fitted regression equations are presented below:


$$\begin{array}{cc} & 𝐓𝐒=-\text{14}.\text{42}+\text{5}.\text{69CA}+\text{0}.\text{44AG}+\text{1}.\text{62EO}-\text{0}.\text{32C}{\text{A}}^{\text{2}}+\text{0}.\text{01A}{\text{G}}^{\text{2}}+\text{0}.\text{48E}{\text{O}}^{\text{2}}-\text{0}.\text{07CA}\\ & \times \text{AG}-\text{0}.\text{13CA}\times \text{EO}-\text{0}.\text{29AG}\times \text{EO} \end{array}$$ (4)

$$\begin{array}{cc} & 𝐄𝐀𝐁=\text{300}.\text{03}-\text{103}.\text{34CA}-\text{6}.\text{62AG}-\text{18}.\text{47EO}+\text{8}.\text{6C}{\text{A}}^{\text{2}}+\text{0}.\text{08A}{\text{G}}^{\text{2}}\;-\text{3}.\text{8E}{\text{O}}^{\text{2}}+\text{1}.\text{2CA}\times \text{AG}\\ & +\text{7}.\text{29CA}\times \text{EO}+\text{0}.\text{04AG}\times \text{EO} \end{array}$$ (5)

$$\begin{array}{cc} & 𝐘𝐌=-\text{156}.\text{95}+\text{63}.\text{30CA}+\text{2}.\text{80AG}+\text{23}.\text{49EO}-\text{3}.\text{17C}{\text{A}}^{\text{2}}-\text{0}.\text{006A}{\text{G}}^{\text{2}}+\text{11}.\text{07E}{\text{O}}^{\text{2}}-\text{0}.\text{62CA}\\ & \times \text{AG}-\text{12}.\text{78CA}\times \text{EO}-\text{0}.\text{75AG}\times \text{EO} \end{array}$$ (6)

**Fig 3:**
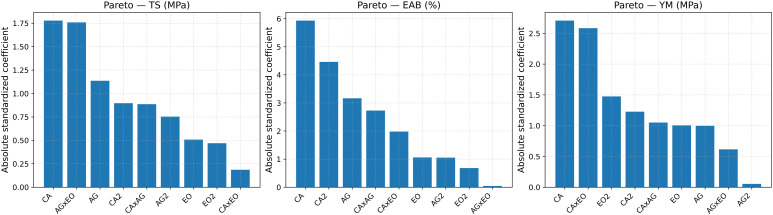
Pareto charts of standardized regression coefficients showing the relative importance of formulation factors and their interactions for (a) TS, (b) EAB, and (c) YM. Terms include linear effects (CA, EO, AG), quadratic effects (CA^2^, EO^2^, AG^2^), and two-factor interactions (CA × EO, CA × AG, EO × AG).

**Fig 4:**
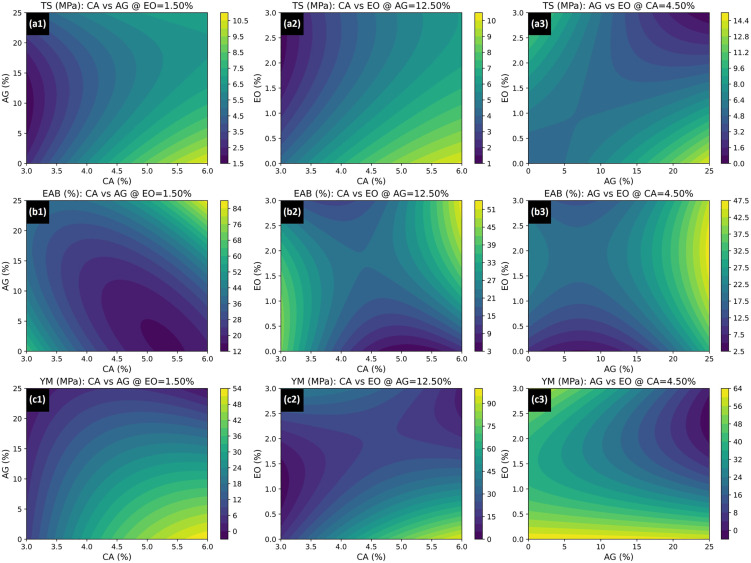
Contour plots showing the combined effects of formulation factors on (a) tensile strength (TS), (b) elongation at break (EAB), and (c) Young’s modulus (YM). Columns represent factor pairs: CA–AG (1), CA–EO (2), and AG–EO (3), with the third variable held constant at its median level.

The model interpretation was based on Pareto charts of absolute standardized regression coefficients (Fig 3), which rank the relative importance of individual terms, and contour plots (Fig 4), which illustrate the combined influence of two variables while holding the third constant at its median level. The models provided an exploratory description of the experimental data across all three mechanical responses. Although the 11 experimental runs were generated using a space-filling Latin Hypercube Design and each mechanical measurement was performed in triplicate, the RSM models were based on 11 unique formulation points; therefore, where applicable, the model outputs were interpreted as exploratory trends rather than definitive causal relationships.

For TS, the model achieved an R^2^ of 0.92 with an adjusted R^2^ of 0.23 and an RMSE of 0.84, indicating that although most of the variability was captured, predictive power was limited due to the small dataset relative to the number of model terms. The model for EAB performed slightly better, with an R^2^ of 0.93, an adjusted R^2^ of 0.29, and an RMSE of 4.42, which represents a smaller relative error when expressed as a fraction of the EAB range, showing a somewhat stronger fit to experimental trends. Therefore, despite their high R² values, the low adjusted R² values indicate that the TS and EAB models should be interpreted cautiously as trend-descriptive rather than strongly predictive. The best performance was obtained for YM, with an R^2^ of 0.99, an adjusted R^2^ of 0.95, and an RMSE of 1.65, suggesting that the model captured most of the variation in stiffness within the investigated design space. Based on the fitted models, regression, ANOVA, and Pareto analyses were used to examine the relative influence of formulation factors on each mechanical property. For TS, both the linear and interaction effects of CA, particularly its interaction with AG, contributed most strongly, while the quadratic effect of CA was more pronounced for EAB. For YM, both CA and EO exhibited major influences, with EO showing a positive contribution to stiffness. These relationships are discussed in detail in the following subsections.

#### 3.2.1. Importance of formulation factors.

The relative influence of the formulation variables and their interactions on the three mechanical responses is illustrated in the Pareto charts of standardized regression coefficients (Fig 3a–c). For TS (Fig 3a), CA was the most influential variable, closely followed by the combined contribution of AG and EO (i.e., the AG × EO interaction), while AG alone also contributed strongly. The quadratic effect (i.e., CA^2^) and CA × AG ranked next in importance, suggesting that strength was shaped not only by CA but also by its combined effects with AG and EO. EO and its quadratic term were less prominent, and CA × EO had only a minor role.

For EAB (Fig 3b), CA was again dominant, with its linear and quadratic terms exerting the largest influence. AG ranked next in importance, followed by CA × AG and CA × EO effects, confirming that extensibility was sensitive to both the concentration of CA and its interplay with the other additives. EO and AG showed moderate contributions, while EO^2^ and AG^2^ had smaller but still notable effects.

In the case of YM (Fig 3c), CA was the strongest factor, followed by CA × EO and the quadratic EO^2^ term. The CA^2^ and CA × AG effects were also substantial, while EO and AG contributed to a lesser extent. The AG × EO and AG^2^ effects were comparatively minor. This pattern indicates that stiffness was primarily controlled by CA and its interactions, with EO playing a secondary but non-negligible role, particularly through its quadratic effect.

These results show that strength (i.e., TS) is determined by CA together with AG and AG × EO synergy, flexibility (i.e., EAB) is governed by CA supported by AG and its interactions, and stiffness (i.e., YM) is mainly driven by CA and CA × EO, with quadratic EO effects also important. This differentiation among responses demonstrates that no single factor dictates film performance, emphasizing the importance of considering both main and interaction terms when designing cellulose acetate films for packaging.

#### 3.2.2. Influence of cellulose acetate (CA).

CA exerted a dominant influence across all three mechanical responses. For TS, CA was the most significant factor in the Pareto analysis (Fig 3a). The CA–AG contours (Fig. 4a1) showed that increasing CA enhanced strength, particularly at moderate AG levels. The CA–EO surfaces (Fig. 4a2) further confirmed that higher CA promoted stronger films, although excessive EO slightly reduced cohesion. This reflects the role of CA as the principal film-forming polymer, reinforcing the matrix and providing load-bearing capacity.

For EAB, both linear and quadratic terms of CA were highly influential (Fig 4b), demonstrating its nonlinear role in film flexibility. The CA–AG plots (Fig. 4b1) indicated that extensibility improved at higher CA when AG was also elevated, whereas low CA contents consistently led to brittle films. The CA–EO plots (Fig. 4b2) showed that extensibility was highest at low CA with low EO; however, when CA was increased, a simultaneous increase in EO was necessary to sustain flexibility. In contrast, high CA with low EO produced stiff films with limited deformability. These patterns highlight that the effect of CA on flexibility depends strongly on its combined formulation with AG and EO.

For YM, CA was again the leading factor (Fig 4c). The CA–AG plots (Fig. 4c1) revealed a steady increase in stiffness with rising CA, confirming its reinforcing effect. The CA–EO surfaces (Fig. 4c2) showed that YM was primarily controlled by CA, with stiffness being highest at elevated CA combined with low EO, while EO itself had little independent effect. This highlights that chain packing and rigidity are predominantly dictated by CA concentration [34,35].

These findings confirm that CA functions as the central structural determinant of film performance. It provides strength and stiffness to the material, while its proportion relative to AG and EO influences flexibility and extensibility. Depending on these interactions, the resulting films may exhibit brittle, extensible, or rigid characteristics. Therefore, precise adjustment of CA content in relation to the other components is essential for tailoring packaging films with targeted mechanical profiles.

The dominant role of CA in strengthening and stiffening the films is consistent with its well-established function as a primary film-forming polymer, wherein CA forms a dense matrix through dipole–dipole interactions among acetyl carbonyl groups, residual hydrogen bonding, and chain entanglement, thereby enhancing load transfer and improving stiffness [36]. Previous studies on CA and other cellulose derivatives have also reported that increasing polymer concentration enhances tensile strength but reduces elongation due to restricted molecular mobility [37–39]. The nonlinear effect observed in this study, where both very low and very high CA contents reduced extensibility, is consistent with findings in CA–chitosan and CA–starch films, where excess CA produced brittle structures with limited deformation capacity [40–42]. These results reinforce the view that CA provides essential structural integrity but requires complementary additives to offset its rigidity. In particular, the interactions with AG and EO identified here suggest a pathway for tailoring CA-based films toward flexible or balanced applications while maintaining sufficient strength for packaging performance.

#### 3.2.3. Influence of aloe vera gel (AG).

AG primarily functioned as a modulator of flexibility within the films. In the Pareto analyses, AG ranked below CA for both TS and YM, but exerted a stronger influence on EAB, where it was identified as the third most significant factor after CA and CA^2^ (Fig 4a–c). This confirms that the major contribution of AG lies in promoting extensibility rather than strength or stiffness.

For TS, AG played a secondary but context-dependent role. The CA–AG contours (Fig. 4a1) indicated that at low-to-moderate CA levels, the effect of AG on TS was not clearly discernible, whereas at high CA, increasing AG reduced TS, consistent with an unfavorable CA × AG effect. The AG–EO surfaces (Fig. 4a3) further highlighted that high AG in combination with high EO markedly weakened TS, pointing to a compounded softening effect.

In the case of EAB, AG again ranked next to CA in importance (Fig 3b). The CA–AG surfaces (Fig. 4b1) showed that higher AG improved extensibility at moderate–high CA levels, mitigating the stiffening effect of CA. The AG–EO plots (Fig. 4b3) further confirmed this behavior, with greater AG enhancing flexibility across most EO concentrations, except when EO was excessive. These results demonstrate the function of AG as a natural plasticizer, though its beneficial effect appears to operate within an optimum range, beyond which the integrity of the film may decline.

For YM, AG acted mainly as a softening factor, in line with its plasticizing role (Fig 4c). The CA–AG contours (Fig. 4c1) revealed that AG slightly counteracted the CA-induced increase in stiffness, particularly when CA was elevated. A similar moderating effect was observed in the AG–EO surfaces (Fig. 4c3), where increasing AG reduced YM even at higher EO contents, though the magnitude of this effect remained modest compared with CA.

Hence, AG can be regarded as a complementary component that enhances flexibility and reduces stiffness without substantially strengthening the films. Its contribution is especially valuable in counterbalancing the rigidifying influence of CA, enabling the design of films that achieve a more desirable balance between extensibility and structural integrity. The plasticizing role of AG observed in this study is in line with previous reports on the use of natural polysaccharide- or gel-based additives in biopolymer films [43–45]. By increasing chain mobility and reducing intermolecular interactions, such additives generally enhance flexibility while lowering stiffness and strength within the polymer network. Similar outcomes have been documented for aloe vera extracts incorporated into starch- and protein-based films, where the bioactive polysaccharides acted as natural plasticizers by introducing additional free volume and interrupting dense hydrogen-bonded networks [46–48]. The diminishing returns observed at very high AG levels, where extensibility declined, are consistent with earlier findings that excessive plasticizer disrupts cohesive interactions, promotes microphase separation, and generates structurally weaker matrices [49,50]. These results suggest that AG is effective in improving the flexibility of CA films, but its concentration must be carefully optimized to avoid compromising mechanical integrity, particularly when combined with other additives such as EO.

#### 3.2.4. Influence of lemongrass essential oil (EO).

EO influenced film performance in a concentration-dependent and interaction-driven manner. In the Pareto analyses, EO contributed moderately to TS and EAB, and more noticeably to YM through interaction and quadratic terms, although CA remained the dominant factor across all responses (Fig 4a–c).

For TS, the CA–EO contours (Fig. 4a2) showed that increasing EO generally reduced strength, an effect most evident at higher CA levels, consistent with the negative CA × EO interaction effect. In AG–EO space (Fig. 4a3), the effect of EO was not pronounced at low AG, whereas at high AG, increasing EO clearly reduced TS, suggesting that the combined softening from both components compromised matrix cohesion.

For EAB, EO had a smaller influence compared with CA or AG (Fig 4b), but its effect was strongly dependent on CA. The CA–EO plots (Fig. 4b2) indicated that extensibility was highest at low CA with low EO, while at higher CA, greater EO was required to sustain EAB. When CA was elevated but EO remained low, films became brittle, whereas higher EO at high CA partially restored deformability. In AG–EO plots (Fig. 4b3), the flexibility-enhancing role of AG was counteracted by EO, with extensibility decreasing when both were simultaneously elevated.

For YM, CA was the primary determinant of stiffness (Fig 4c). The CA–EO surfaces (Fig. 4c2) demonstrated that YM rose steeply with CA and reached its maximum at high CA with low EO, while increasing EO under high CA conditions reduced YM. In AG–EO surfaces (Fig. 4c3), the effect of EO was limited at low AG (YM appeared higher but likely reflected the fixed CA concentration of 4.5%) whereas at high AG, a clear decline in YM occurred with increasing EO. This indicates that EO contributed to softening when combined with elevated AG, consistent with its role as a plasticizing additive at higher concentrations.

EO, therefore, acted as a conditional modifier. At low to moderate levels, it had limited influence; while at higher concentrations it reduced strength and extensibility, and softened the films in the presence of AG. Its effects were nonlinear and dependent on CA and AG, highlighting the need for careful tuning of EO content in the formulation to achieve the desired balance of mechanical properties. The dual role of EO, acting as a plasticizer at lower concentrations and as a disruptive or stiffening agent at higher levels, reflects trends reported for other essential oils in biopolymer films [51,52]. At moderate contents, EO molecules may intercalate between polymer chains, reducing intermolecular forces and enhancing chain mobility, thereby contributing to limited flexibility [53]. However, at elevated levels, the hydrophobic nature of EO can induce phase separation or microstructural discontinuities, weakening cohesive strength and altering stiffness [54]. Similar behavior has been noted in starch- and chitosan-based films containing essential oils, where mechanical performance was highly sensitive to concentration and interactions with the matrix [55,56]. The present results confirm that EO exerts only a secondary influence relative to CA and AG, but its conditional effects make it an important factor for fine-tuning performance, particularly when high CA or AG levels are present.

#### 3.2.5. Interaction effects and trade-offs (Contour plot analysis).

The contour plots (Fig 4) provide a comprehensive view of how pairs of formulation factors interact to determine the mechanical behavior of the films, with the third factor held at its median level. The results demonstrate that film properties are not governed by any single component in isolation; rather, the overall mechanical response arises from the combined influence of CA, AG, and EO. CA remained the primary determinant of strength and stiffness, AG functioned mainly as a plasticizer enhancing extensibility, and EO acted as a conditional modifier (reducing TS and YM when present at high levels alongside CA or AG), but exerting a mild stiffening effect at lower CA concentrations. These patterns highlight the complex interplay among formulation variables that defines the mechanical profile of the films.

The combined effects observed here reflect a broader design challenge common to biopolymer-based films: improving one mechanical attribute often compromises another. Similar trade-offs among tensile strength, elongation, and stiffness have been widely reported for starch-, protein-, and cellulose-derived systems, where the incorporation of plasticizers or bioactive agents shifts the balance between cohesion and flexibility. For instance, increasing polymer or reinforcing agent content typically enhances strength and stiffness but reduces elongation [57–60], whereas plasticizers and natural extracts improve extensibility at the expense of strength. In this context, the ability of AG and EO to partially counteract the rigidifying effect of CA demonstrates how compositional tuning can yield balanced formulations without relying on multilayer or composite designs. These interdependencies emphasize the complex balance between formulation variables, where improving one property often compromises another. This behavior formed the basis for the classification framework developed in Section 3.3, in which films were categorized as flexible, balanced, or rigid according to their mechanical performance profiles.

### 3.3. Classification of films into flexible, balanced, and rigid types

To translate the mechanical performance of the films into practical design guidance, the results of experimental testing and RSM predictions were classified into three functional categories: flexible, balanced, and rigid. This classification was established using a specification-driven approach, where threshold values for TS, EAB, and YM were derived from the observed ranges of the experimental dataset. By applying these criteria, the films could be systematically linked to intended packaging functions, with flexible films suited for wrapping and sealing applications, rigid films for structural or protective uses, and balanced films for multipurpose applications requiring both strength and extensibility.

Flexible films were defined by high extensibility (EAB ≥ 40%), low stiffness (YM ≤ 20 MPa), and moderate strength (TS ≥ 3 MPa). Rigid films were characterized by high strength (TS ≥ 8 MPa) and high stiffness (YM ≥ 50 MPa), together with low extensibility (EAB ≤ 20%). Balanced films occupied the intermediate range (TS 3.5–6.5 MPa, EAB 20–40%, YM 20–50 MPa). The adopted classification rules are summarized in Table 2. Strict classification was applied only when all three property conditions were met simultaneously.

**Table 2:** The criteria used for classification of films into flexible, balanced, and rigid categories.

Category	Tensile strength (TS)	Elongation at break (EAB)	Young’s modulus (YM)
Flexible	TS ≥ 3 MPa	EAB ≥ 40%	YM ≤ 20 MPa
Balanced	TS = 3.5–6.5 MPa	EAB = 20–40%	YM = 20–50 MPa
Rigid	TS ≥ 8 MPa	EAB ≤ 20%	YM ≥ 50 MPa

Note: A film was assigned to a category only when all three mechanical criteria for that category were simultaneously satisfied.

**Fig 5:**
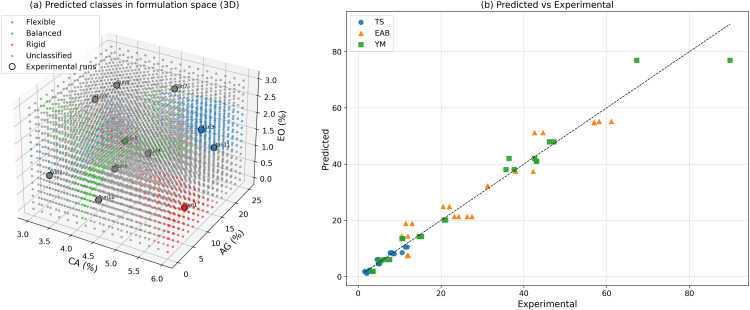
(a) Predicted classification map of formulation space showing flexible, balanced, and rigid regions with experimental runs overlaid. (b) Fitted predicted values versus experimental values for TS, EAB, and YM with a 1:1 reference line.

The distribution of categories across the formulation space is illustrated in Fig 5a, showing the predicted regions for flexible, balanced, and rigid films together with experimental runs overlaid. The flexible domain was located primarily at higher EAB and lower YM predictions, within which Run 3 and Run 11 were identified as flexible. Flexible behavior was generally associated with higher AG concentrations combined with moderate CA and EO levels, conditions that enhanced extensibility while minimizing stiffness. The rigid region was predicted at high TS and YM values, with Run 1 classified as rigid. These formulations corresponded to CA-rich compositions, where elevated polymer content reinforced strength and stiffness but restricted deformability. Run 9 was the only formulation that fully met the criteria for the balanced category, exhibiting moderate tensile strength, extensibility, and stiffness within the defined specification ranges. Beyond this, several other formulations could not be strictly classified and were designated as unclassified. These included Runs 2, 4, 5, 6, 7, 8, and 10, which displayed partial alignment with one or more class-defining properties but did not satisfy all three criteria required for assignment to the flexible, balanced, or rigid groups. This mapping highlights how the adopted classification scheme differentiates the experimental runs across the design space. Fig 5b further shows the agreement between fitted predicted values and experimental values within the same dataset, supporting the descriptive use of the models described in Section 3.2.

From a practical standpoint, classifying films into flexible, balanced, and rigid categories provides a direct link between mechanical performance and packaging functionality. However, this framework should be considered an initial mechanical screening tool, and its practical utility requires further validation through real packaging trials and functional performance testing. To the best of our knowledge, there are limited reports that propose quantitative classification frameworks or standardized thresholds for active biopolymer-based packaging films. Establishing such criteria, as done in this study, offers a valuable step toward aligning mechanical testing outcomes with real-world packaging applications. By defining clear mechanical ranges, this approach bridges the gap between experimental characterization and performance-based design, highlighting that no single formulation can satisfy all requirements simultaneously. Instead, tailoring films to specific use cases ensures functionality while preserving the sustainability advantages of bio-based materials.

## 4. Conclusion

This study demonstrated that the mechanical performance of cellulose acetate films can be systematically tailored through the incorporation of aloe vera gel and lemongrass essential oil. By integrating experimental testing with statistical modeling, we identified the dominant role of CA in providing strength and stiffness, the plasticizing effect of AG in enhancing extensibility, and the conditional influence of EO as a secondary modifier. The interaction analyses revealed clear trade-offs, showing that improvements in one property often came at the expense of another, highlighting the necessity of balancing formulation components. Importantly, a specification-driven classification framework was applied to categorize the films into flexible, balanced, and rigid types based on defined thresholds for tensile strength, elongation at break, and Young’s modulus. Using these criteria, Run 3 and Run 11 were identified as flexible, Run 9 as balanced, and Run 1 as rigid, while several other formulations remained unclassified due to partial compliance. This classification approach directly linked mechanical profiles to potential packaging applications, with flexible films suited for wrapping, rigid films for protective structures, and balanced films for multipurpose uses. These findings highlight how data-driven classification can support the rational design of bioactive CA films, enabling the development of bio-based packaging materials tailored to specific functional performance requirements. Future work should include functional, structural, thermal, and real packaging evaluations to further validate their practical application potential.
